# Supplementary material for: The Golgi Glycoprotein MGAT4D is an Intrinsic Protector of Testicular Germ Cells From Mild Heat Stress
Source: Sci Rep. 2020 Feb 7;10:2135. doi: 10.1038/s41598-020-58923-6 (PMC7005853; doi:10.1038/s41598-020-58923-6)
Supplement: Supplementary file 1 — Supplementary information [file 41598_2020_58923_MOESM1_ESM.pdf]

Supplementary Material

The Golgi Glycoprotein MGAT4D is an Intrinsic Protector of Testicular Germ Cells From Mild Heat Stress

Ayodele Akintayo[1]*, Meng Liang[1,3]*, Boris Bartholdy[1], Frank Batista[1], Jennifer Aguilan[2], Jillian Prendergast[1,4], Afsana Sabrin[1], Subha Sundaram[1], and Pamela Stanley[1]

[1] Dept. Cell Biology, Albert Einstein College Medicine, New York, NY, United States

[2] Laboratory for Macromolecular Analysis and Proteomics Facility, Dept. Pathology, Albert Einstein College Medicine, New York, NY, 10461

Supplementary Methods

MALDI-IMS. Male mice were sacrificed, testes dissected, decapsulated, fixed in Bouin's fixative and embedded in paraffin. Sections of 6 µm were cut and mounted on Indium tin oxide (ITO) glass slides (Delta Technologies, Ltd., CO). Slides were deparaffinized and antigen retrieval was performed using citraconic anhydride buffer at pH 3 in a steamer for 30 min. Aqueous solution of PNGase F Prime (0.1 µg/µl, N-Zyme Scientifics, LLC, PA) was spray-coated on the slides using a TM Sprayer (HTX Technologies, LLC, NC) [1]. Slides were incubated at 37°C overnight in a humidified chamber and dried in a vacuum desiccator. Slides were then coated with α-cyano-4-hydroxycinnamic acid (CHCA) matrix (7 mg/ml in 50% Acetonitrile/ 0.1% TFA) using the TM Sprayer (HTX Technologies, LLC, NC). N-glycan imaging was performed by scanning and acquiring spectra from the entire tissue sections (m/z range 900-4,500) on an Ultraflextreme MALDI-TOF/TOF mass spectrometer equipped with a SmartBeam II laser at 1 kHz and 100 µm raster width. MALDI-TOF data were processed by FlexImaging software (v 4.1) to generate N-glycan ion maps.

Confocal Microscopy

Testes from 7-month males were fixed in Bouin's fixative (#100503-962, Electron Sciences, Radnor, PA, USA) and paraffin-embedded by the Einstein Histology and Comparative Pathology Core Facility. Unstained sections (5 µm) were collected on positively-charged slides for immunofluorescent staining. Histoclear (HS-200, National Diagnostics, Atlanta, GA) was used for deparaffinization followed by heat-induced epitope retrieval in citrate buffer (10 mM sodium citrate with 0.05% Tween-20, pH 6.0) for 20 min at 100ºC. Permeabilization was performed using 0.1% Triton X-100 in Tris-buffered saline

(TBS) for 10 min and the samples were blocked with 10% serum (from the same species as secondary antibody) for 2 hr at room temperature and incubated overnight at 4°C with primary antibodies in TBS 1% BSA - mouse monoclonal antibody (mAb) GM130 (#610822 BD Transduction Laboratories) and anti-MGAT4D C-terminus pAb (Genemed, Torrance, CA). After rinsing with TBS containing 0.025% Triton X-100, sections were incubated with Alexa fluor 488 goat anti-mouse IgG (H+L) (Invitrogen) and Alexa fluor 594 chicken anti-rabbit IgG (H+L) (Molecular Probes, Oregon, USA) in TBS 1% BSA to detect GM130 and MGAT4D, respectively. Hoechst stain 33342 (Molecular Probes, Oregon, USA) was used to counterstain the sections followed by mounting in ProLong Diamond Antifade Mountant (#P36961, Molecular Probes, Oregon, USA). Images were obtained using Leica SP8 confocal microscope (SIG #1S10OD023591-01) at the Einstein Analytical Imaging Facility.

References

1. Black, A. P. *et al.* A Novel Mass Spectrometry Platform for Multiplexed N-Glycoprotein Biomarker Discovery from Patient Biofluids by Antibody Panel Based N-Glycan Imaging. *Anal Chem* **91**, 8429-8435, doi:10.1021/acs.analchem.9b01445 (2019).
2. Neelamegham, S. *et al.* Updates to the Symbol Nomenclature for Glycans guidelines. *Glycobiology* **29**, 620-624, doi:10.1093/glycob/cwz045 (2019).
3. Varki, A. *et al.* Symbol Nomenclature for Graphical Representations of Glycans. *Glycobiology* **25**, 1323-1324, doi:10.1093/glycob/cwv091 (2015).
4. Varki, A. *et al.* Symbol nomenclature for glycan representation. *Proteomics* **9**, 5398-5399, doi:10.1002/pmic.200900708 (2009).
5. Huang, H. H. *et al.* GnT1IP-L specifically inhibits MGAT1 in the Golgi via its luminal domain. *Elife* **4**, doi:10.7554/eLife.08916 (2015).
6. Huang, H. H. & Stanley, P. A testis-specific regulator of complex and hybrid N-glycan synthesis. *J Cell Biol* **190**, 893-910, doi:10.1083/jcb.201004102 (2010).

Supplementary figure legends

Figure S1. Full western blot for Fig. 1C. Germ cell extracts from males of the designated genotypes were analyzed by SDS PAGE, transferred to membrane and western blot analysis was performed with an antigen-purified, pAb to a C-terminal peptide of MGAT4D.

Figure S2. MALDI-IMS analysis of wild type and *Mgat4d*[-/-] testis sections. (**A**) Representative images of testis sections showing, in pseudo-color, the relative abundance of the predicted N-glycan shown on the left of each row. (**B**) MALDI-IMS intensity profiles of predicted N-glycans in *Mgat4d*[+/+] (top) and *Mgat4d*[-/-] (bottom) testis sections. (**C**) Graphical representation of the spectrum mean intensity ratio in testis sections from *Mgat4d*[-/-] (n=4) to *Mgat4d*[+/+] (n=3) for each N-glycan species. ***p<0.001 based on unpaired, two-tailed Student's t test assuming equal STDEV. (**D**) Graphical representation of the mean intensity ratio for testis sections from transgenic *Stra8-Mgat4d-Myc/Ldhc-Mgat4d-Myc* (n=3) or *Stra8-Mgat4d-Myc/Ldhc-Mgat4d-Myc/Prm1-Mgat4d-Myc* (n=2) transgenic mice over *Mgat4d*[+/+] (n=3) for each N-glycan species. N-glycans are represented using the Symbol Nomenclature for Glycans [2-4]; GlcNAc, blue square; Man, green circle, Gal, yellow circle; Fuc. red triangle; Sia, red diamond.

Figure S3. Characterization of antibodies against MGAT4D and confocal microscopy. (**A**) Extracts from CHO transfectants stably-expressing Myc-tagged MGAT4D [5,6] were subjected to western blot analysis using pAbs raised in rabbits against the N-terminus unique to MGAT4D-L. (**B**) The same CHO extracts were subjected to western analysis using rabbit pAbs against a C-terminal peptide of MGAT4D (lanes 1,2). Myc-MGAT4D-L had a much stronger signal than MGAT4D-L-Myc, presumably because the Myc sequence perturbed the epitope. The pAb detected endogenous MGAT4D-L and MGAT4D-S in lane 4 in wild type germ cells, and those signals were absent from *Mgat4d*[-/-] germ cells in lane 6. Germ cell extracts from 28 dpp males expressing the *Stra8-Mgat4d-L-Myc* and *Ldhc-Mgat4d-L-Myc* transgenes (lane 8) or the *Ldhc-Mgat4d-L-Myc* transgene alone (lane 9) exhibited endogenous signals as expected, but no clear transgene signal. (**C**) Confocal microscopy of testis sections from wild type with Abs to GM130 and the C-terminus of MGAT4D. The yellow square is the area enlarged in the bottom row of images. Scale bar 20 μm. The signal for MGAT4D overlaps with the signal for the GM130 Golgi marker in all cells where MGAT4D is expressed.

Figure S4. Evidence of *Mgat4d* exon 4 excision. (**A**) Diagram representing exons in the mouse showing primers (A, B, C, D) used for PCR of germ cell cDNA. (**B**) Diagram extracted from TAC4 software representing *Mgat4d* exon transcript abundance in germ cell cDNA from *Mgat4d*[-/-] (KO) and *Mgat4d*[+/+] (WT). Exon 4 has a low signal in the KO preparation. Other exons are transcribed explaining why *Mgat4d* is represented in *Mgat4d*[-/-] microarray data. However, no protein was observed from these transcripts (Fig. 1).

Figure S5. Gene set enrichment analysis. (**A**) Top enriched Hallmark gene sets in *Mgat4d*[-/-] germ cells obtained from Gene Set Enrichment Analysis (GSEA) with Normalized Enrichment Scores (NES) as shown. Each gene set had a nominal *p* value <0.05 and FDR <25%. (**B**) Top enriched Hallmark gene sets in *Mgat4d*[+/+] germ cells with NES shown. Each gene set had a nominal *p* value <0.05 and FDR <25%. (**C**) Top section of the heat map of genes from the Hallmark spermatogenesis gene set enriched in *Mgat4d*[-/-] germ cells. (**D**) Top section of the heat map of genes from the Hallmark gene set TNF alpha signaling via NFκB enriched in *Mgat4d*[+/+] germ cells.

Supplementary Table S1. Genotyping Primers

**Gene or Transgene**	**Primer name**	**Sequence**	**Product length (bp)**
Mgat4d floxed	FB77-Fw<br>FB78-Rev	TCCCACCCATGAAACAGTCT<br>GTACTGGAGCCCAAGCAGAA	477 *Mgat4d*[+]<br>623 *Mgat4d*[F]
Mgat4d deleted	FB79-Fw<br>FB-80-Rev	CCAGAGCTTAGAAAGCTGGTGT<br>TCAGTAATGGCTTTAATGGTCTATTT	1008 *Mgat4d*[+]<br>246 *Mgat4d*[-]<br>1152 *Mgat4d* [F]
Stra8-iCre	Stra-Fw<br>Stra-Rev	AGATGCCAGGACATCAGGAACCTG<br>ATCAGCCACACCAGACACAGAGATC	236
Stra8-Tg junction	Stra8-*Mgat4d*-Fw<br>*Mgat4d*-Rev	CATCTTGCTCCTTCCACACCC<br>TGGAGATGCAGAAACACGAGAAACC	639
Ldhc-Tg junction	Ldhc-*Mgat4d*-Fw<br>*Mgat4d*-Rev	TGGAAACCGTCTGGAGTCGT<br>TGGAGATGCAGAAACACGAGAAACC	536
Prm1-Tg junction	Prm1-*Mgat4d*-Fw<br>*Mgat4d*-Rev	TGAAGCACTTGATGGGGCCT<br>TGGAGATGCAGAAACACGAGAAACC	857

Supplementary Table S2. Testis weights.

Mouse Background	Temp. (°C)	Genotype	Testis Weight (mg) (+/- SEM)	Testis/Body weight (+/- SEM)
FVB	33	*Mgat4d*[-/-] (n=4)	81.7 (1.2)	0.002 (8.8E-05)
	43	*Mgat4d*[+/-] (n=2)	71.5 (14.8)	0.0017 (2.3E-04)
		Mgat4d[-/-] (n=5)	71.5 (3.9)	0.0015 (4.0E-05)
C57Bl6/J	33	*Mgat4d*[+/+] (n=3)	90.4 (3.6)	0.0031 (9.4E-05)
		Stra8-*Mgat4d-Myc* (n=3)	99.3 (3.3)	0.0031 (1.4E-04)
		Ldhc-*Mgat4d-Myc* (n=4)	96.8 (2.7)	0.0026 (9.8E-05)
		Prm1-*Mgat4d-Myc* (n=3)	106.3 (2.7)	0.0030 (7.4E-04)
	43	*Mgat4d*[+/+] (n=5)	85.4 (1.1)	0.0026 (3.2E-05)
		Stra8-*Mgat4d-Myc* (n=11)	86.6 (2)	0.0025 (4.5E-05)
		Ldhc-*Mgat4d-Myc* (n=7)	88.1 (2)	0.0024 (6.5E-05)
		Prm1-*Mgat4d-Myc* (n=5)	94.5 (3.6)	0.0029 (1.6E-04)

Supplementary Table S3. DEGs for *Mgat4d*[-/-] versus *Mgat4d*[+/+] treated at 33°C ($p<0.05$; Log_2 Fold change +/-0.585 ; FDR<0.05).

**Gene_id**	**REFSEQ**	**Gene Symbol**	**Log_2 Fold Change**	**P.Value**	**FDR P.Val**
ENSMUSG00000035057.7	NM_026233.2;NA	*Mgat4d*	-2.476254706	1.71199E-12	7.07687E-08
ENSMUSG00000083857.1	N/A	Gm12584	0.651408486	6.05467E-06	0.031285232
ENSMUSG00000096243.1	N/A	Gm24265	0.59577105	1.36822E-05	0.035348888
ENSMUSG00000031919.6	NM_025458.2	*Tmed6*	0.615438203	2.11808E-05	0.04457681

N/A, none available

Supplementary Table S4 Top 20 Down-regulated genes in *Mgat4d*[-/-] vs *Mgat4d*[+/+] treated at 43°C ($p<0.0$; Log_2 Fold change +/-0.585; FDR<0.05).

**Gene_id**	**REFSEQ**	**Gene Symbol**	**Log_2 Fold Change**	**P.Value**	**FDR P.Val**
ENSMUSG00000044734.16	NM_025429.2;NA	*Serpinb1a*	-2.13291396	1.62393E-08	0.000466018
ENSMUSG00000112023.1	NM_001291892.1;NM_008147.2;NA; NM_001291893.1	*Ly96*	-2.127378416	7.72306E-07	0.001596241
ENSMUSG00000029561.17	NM_011854.2;NA	*S100a11*	-2.095241568	5.88104E-05	0.009529246
ENSMUSG00000107988.1	NM_011485.5	*Star*	-1.991773919	0.000273218	0.01981437
ENSMUSG00000001173.15	NM_177215.3;NA	*Gm5552*	-1.946398486	6.5394E-06	0.003790091
ENSMUSG00000021843.17	NM_008477.2;NA;NM_001293636.1; NM_001293635.1;NM_001347522.1	*Osr2*	-1.886229424	7.99092E-06	0.004078031
ENSMUSG00000024697.3	NM_008137.4	*Klk1b22*	-1.757911947	0.000205567	0.017063269
ENSMUSG00000021025.8	NM_010907.2;NA	*Gstm2-ps1*	-1.66489437	0.000239868	0.018637979
ENSMUSG00000115207.1	N/A	*Hspd1-ps5*	-1.649714912	0.000489712	0.025072341
ENSMUSG00000058427.10	NM_009140.2;NA	*Vmn2r-ps55*	-1.561583539	0.000323231	0.021174979
ENSMUSG00000020120.15	NA;NM_019549.2	*Gm906*	-1.512253739	8.96726E-06	0.004310228
ENSMUSG00000107737.1	N/A	*AC113125.1*	-1.501234542	8.60716E-05	0.01161002
ENSMUSG00000075538.2	N/A	*Ly6e-ps1*	-1.471201428	4.64307E-05	0.008418001
ENSMUSG00000102555.1	N/A	*Gm47657*	-1.463936317	2.64999E-06	0.002625423
ENSMUSG00000021109.13	NM_001313919.1;NM_010431.2; NM_001313920.1;NA	*Cxcl1*	-1.432819232	2.97223E-06	0.002681799
ENSMUSG00000032420.8	NM_011851.4;NA	*Cd36*	-1.421730882	0.000385586	0.022681955
ENSMUSG00000020044.13	NM_011595.2;NA	*Gm25992*	-1.420700907	0.000153868	0.015183827
ENSMUSG00000025779.10	NM_016923.2;NM_001159711.1;NA	*Serpina3g*	-1.414715206	4.10519E-05	0.007713469
ENSMUSG00000019850.11	NM_009397.3;NM_001166402.1;NA	*Cyp11a1*	-1.403700796	0.001239342	0.04030738
ENSMUSG00000028128.13	NM_010171.3;NA	*Gm24613*	-1.38644583	0.001591758	0.045661687

N/A, none available

Supplementary Table S5. Top 20 Up-regulated genes for *Mgat4d*[-/-] vs *Mgat4d*[+/+] treated at 43°C (p<0.05; Log_2 Fold change +/-0.585; FDR<0.05).

**Gene_id**	**REFSEQ**	**Gene Symbol**	**Log_2 Fold Change**	**P.Value**	**FDR P.Val**
ENSMUSG00000044734.16	NM_025429.2;NA	*Gm26715*	2.039467879	6.67287E-05	0.01021617
ENSMUSG00000112023.1	NM_001291892.1;NM_008147.2; NA;NM_001291893.1	*Gm48565*	1.977932812	0.000937941	0.034380536
ENSMUSG00000029561.17	NM_011854.2;NA	*Hspa1a*	1.809603885	0.000293976	0.020389374
ENSMUSG00000107988.1	NM_010478.2	*Hspa1b*	1.767645726	0.000438609	0.024104592
ENSMUSG00000001173.15	NM_177215.3;NA	*Gm37692*	1.325226203	9.74602E-05	0.012430179
ENSMUSG00000021843.17	NM_008477.2;NA;NM_001293636.1; NM_001293635.1;NM_001347522.1	*Gm9176*	1.159699663	0.000549294	0.026232849
ENSMUSG00000024697.3	NM_008137.4	*4933414I15Rik*	1.115823396	0.000122704	0.013705122
ENSMUSG00000021025.8	NM_010907.2;NA	*Gm5795*	1.066409818	0.001758011	0.048433599
ENSMUSG00000115207.1	N/A	*Slc13a5*	1.02736092	7.09263E-05	0.010437146
ENSMUSG00000058427.10	NM_009140.2;NA	*Gm5134*	1.026790159	0.000453311	0.024424745
ENSMUSG00000020120.15	NA;NM_019549.2	*Gdpd4*	1.022429475	0.000182972	0.016250887
ENSMUSG00000107737.1	N/A	*Gm37432*	1.01770496	5.5473E-05	0.009209182
ENSMUSG00000075538.2	N/A	*Catspere1*	0.95199565	0.001811793	0.049246727
ENSMUSG00000102555.1	N/A	*Rnf144b*	0.947574235	0.000356009	0.022238592
ENSMUSG00000021109.13	NM_001313919.1;NM_010431.2; NM_001313920.1;NA	*Gm27317*	0.941739767	1.34066E-05	0.005263451
ENSMUSG00000032420.8	NM_011851.4;NA	*Gm19224*	0.939180805	0.000708318	0.029695455
ENSMUSG00000020044.13	NM_011595.2;NA	*Gm26785*	0.925613481	3.87817E-05	0.007526383
ENSMUSG00000025779.10	NM_016923.2;NM_001159711.1;NA	*Cdkl4*	0.91217717	0.000921422	0.034121523
ENSMUSG00000019850.11	NM_009397.3;NM_001166402.1;NA	*n-R5s40*	0.907045248	0.000270505	0.01981437
ENSMUSG00000028128.13	NM_010171.3;NA	*Sgpp2*	0.904271954	0.000321063	0.021167112

N/A, none available

Supplementary Table S6. Quantitative RT-PCR Primers

Gene	Gene ID	Primer name	cDNA (bp)	Sequence
Mgat4d-L	NM_026233.2<br>HM067443	Long-Fw<br>Long-Rev	129	TGCCTGGGAGAAAGTGTTGGGGACC<br>CGTGGCAGCGTCACTGCCAACACCA
Mgat4d-Myc transgene		Tr-Fw<br>Tr Rev	124	GGATTTCGAATTCAGTACAGACCAT<br>CAGATCTTCTTCAGAAATAAGTTTTTGTTC
Star	NM_011485.5	Star-Fw<br>Star-Rev	155	TCCTCGCTACGTTCAAGCTG<br>ACGTCGAACTTGACCCATCC
Osr2	NM_054049.2	Osr2-Fw<br>Osr2-Rev	122	ACATATGCAGACATCAAGCCCT<br>CCTGGGCTTCGCTAGAAGTT
Serpinb1a	NM_025429.2	Serp-Fw<br>Serp-Rev	154	GGCTTTTGCATGACCTCCAG<br>GGCTTAAGGGTATCCACGCT
Cyp11a1	NM_019779.4	Cyp11-Fw<br>Cyp11-Rev	164	GGTTTGGGGCAGAGACACTC<br>AGGTACCAGCTCCCTTTCCA
Ly96	NM_001159711.1	Ly96-Fw<br>Ly96-Rev	145	TGCAACTCCTCCGATGCAAT<br>TACGCTTCGGCAACTTTGGA
KlK1b22	NM_010114.2	Klk1b-Fw<br>KlK1b-Rev	143	TGTCCATCAAGCTCCATCCT<br>ACCATCACAGATCAGTGGGC
Tmed6	NM_025458.2	Tmed6-Fw<br>Tmed6-Rev	158	TCACCTGCAGAAGAACCCAC<br>TCATTCCGATCAGCTCCACG
Slc2a3	NM_011401.4	Slc2a-Fw<br>Slc2a-Rev	174	ACCACGAGGAGGATGTGGTAA<br>AATCTCTGCAAGGGGTGGAG
Gdpd4	NM_177696.3	Gdpd-Fw<br>Gdpd-Rev	149	TGGTCGCCTAGGCTCATAGA<br>GCATTAGGGAGCAACCCACT
Prss42	NM_153099.1	Prss-Fw<br>Prss-Rev	325	CCTCTTGCTCCTTCAGCCAA<br>AATACAATGGGCGGCAGTCA
Pabpc6	NM_001163836.1	Pabp-Fw<br>Pabp-Rev	194	GAGCCGTAGGGCATACTGTG<br>ATGTGCTGGCAGTACGATGT
Hspa1a	NM_010479.2	Hspa1a-Fw<br>Hspa1a-Rev	183	CGAGGAGGTGGATTAGAGGC<br>AGCCCACGTGCAATACACAA

Hspa1b	NM_010478.2	Hspa1b-Fw<br>Hspa1b-Rev	176	ATCAGTGGGCTGTACCAGGG<br>CCAAGCAGCTATCAAGTGCAA
Crybg3	NM_174848.3	Cryb-Fw<br>Cryb-Rev	134	GGCTGCCCATCAGCTAGAAT<br>AAAACGGAATTCACGGCGTC
Hsp90aa1	NM_010480.5	Hsp90-Fw<br>Hsp90-Rev	103	CGAAGCATAACGACGATGAGC<br>ACCTTTGTTCCACGACCCAT
Egfr	NM_007912.4	Egfr-Fw<br>Egfr-Rev	154	GAAGTGTGGCCATCTGGGTA<br>CAGGGCAAGAGGGCAGAATC
Hist1h2aa	NM_175658.2	Hist1-Fw<br>Hist1-Rev	147	GCCAAGGGAACTACGCACAA<br>GCAGGTGGCGAGGAGTAATG
Selenop	NM_001042613.2	Selen-Fw<br>Selen-Rev	152	AACTCGTCAAAAGTCGTCCGT<br>CTATGTACCACTCCGGGGCT
Dnaic2	NM_001034878.3	Dnaic-Fw<br>Dnaic-Rev	172	AAGACCTGGCAAAAAGAGGGAA<br>GAAGGCAAGGTGCTTGGAGG
Bcl2l12	NM_029410.3	Bcl2l12-Fw<br>Bcl2l12-Rev	124	TCTTCTCTAGCCGGGAAAGC<br>CGGCTCAATTCCATGGCTAGT
Degs1	NM_007853.5	Degs1-Fw<br>Degs1-Rev	116	CGAGAGGAGTTCGAATGGGTC<br>CAGATCAGGTTGTGGTCAGGT
Dmrt1	NM_015826.5	Dmrt1-Fw<br>Dmrt1-Rev	129	TACTCAGAAGCCAAAGCCAGT<br>GGACGCAGACTCACATTCCAG
Socs3	NM_007707.3	Socs3-Fw<br>Socs3-Rev	110	CAAGGCCGGAGATTTCGCTT<br>GGAGCCAGCGTGGATCTG
Rps2	NM_008503.5	Rps2-Fwr<br>Rps2-Rev	112	CTGACTCCCGACCTCTGGAAA<br>GAGCCTGGGTCCTCTGAACA
Actb	NM_007393.5	b-Act-Fwr<br>b-Act-Rev	195	GGCTCCTAGCACCATGAAGAT<br>TAAAACGCAGCTCAGTAACAGTC

Supplementary Table S7. Top upstream regulators at 43ºC.

Upstream Regulator	Expr Log Ratio	Molecule Type	Predicted State	Activation z-score	Flags	p-value of overlap	Mechanistic Network
Lipopolysaccharide*		chemical drug	Inhibited	-5.4	bias	1.5E-20	114 (15)
dexamethasone		chemical drug		-1.8		8.2E-20	136 (17)
TGFB1	-0.507	growth factor	Inhibited	-3.3		3.8E-19	126 (17)
TNF	-0.379	cytokine	Inhibited	-3.8		3.9E-19	126 (17)
IFNG		cytokine	Inhibited	-4.3		1.0E-18	124 (17)
IL4		cytokine		-1.9	bias	6.0E-18	114 (15)
IL1B		cytokine	Inhibited	-3.9		5.1E-17	125 (14)
dihydrotestosterone		chemical - endogenous mammalian	Inhibited	-2.2	bias	2.5E-16	124 (20)
forskolin		chemical toxicant	Inhibited	-3.7	bias	3.7E-16	133 (24)
tetradecanoylphorbol acetate		chemical drug	Inhibited	-5.3	bias	4.7E-16	144 (22)
AGT	-1.091	growth factor	Inhibited	-3.8	bias	8.8E-15	118 (17)
SB203580		chemical - kinase inhibitor	Activated	2.9	bias	1.3E-14	123 (18)
cigarette smoke		chemical toxicant	Inhibited	-3.0	bias	2.0E-14	118 (22)
tretinoin		chemical - endogenous mammalian	Inhibited	-3.8		2.6E-14	133 (19)
NFKBIA	-1.104	transcription regulator		-1.5		2.9E-14	118 (17)
STAT6		transcription regulator		0.6		5.0E-14	119 (15)
NR3C1		ligand-dependent nuclear receptor		0.9		8.1E-14	141 (20)
NFE2L2		transcription regulator	Inhibited	-2.8	bias	9.9E-14	89 (15)
beta-estradiol		chemical - endogenous mammalian		-1.9		1.9E-13	115 (15)
EGF		growth factor	Inhibited	-3.2	bias	2.5E-13	131 (22)
LY96#	-2.127	transmembrane receptor				7.3E-04	108 (20)
STAR	-1.992	transporter				7.9E-03	
OSR2	-1.886	transcription regulator				3.4E-02	
CXCL2	-1.433	cytokine		-1.1	bias	2.7E-05	79 (13)
ANXA5	-1.332	transporter				7.6E-04	46 (7)
IL1A	-1.313	cytokine	Inhibited	-2.7	bias	1.7E-09	91 (12)
F3	-1.229	transmembrane receptor				4.3E-02	
NFKBIA	1.104	transcription regulator		-1.5		2.9E-14	118 (17)
AGT	-1.091	growth factor	Inhibited	-3.8	bias	8.8E-15	118 (17)
FXYD1	-1.081	ion channel				1.1E-02	
HPGD	-1.07	enzyme				2.3E-02	
SLC13A5	1.027	transporter				4.5E-02	
HSPA1A/HSPA1B	1.81	enzyme				3.8E-04	64 (7)

* following sorted by p-value of overlap
\# following sorted by expression log ratio

Supplementary Table S8. Top Networks in *Mgat4d*[-/-] versus *Mgat4d*[+/+] at 43°C.

Rank	Score	Focus Molecules	Top Diseases and Functions
1	52	28	[DNA Replication, Recombination, and Repair, Nucleic Acid Metabolism, Small Molecule Biochemistry]
2	30	19	[Cell Death and Survival, Cellular Compromise, Endocrine System Development and Function]
3	26	17	[Lipid Metabolism, Molecular Transport, Small Molecule Biochemistry]
4	26	17	[Cell-To-Cell Signaling and Interaction, Cellular Movement, Tissue Development]
5	24	16	[Carbohydrate Metabolism, Cellular Compromise, Small Molecule Biochemistry]
6	22	15	[Cardiovascular Disease, Drug Metabolism, Glutathione Depletion In Liver]
7	20	14	[Cardiovascular System Development and Function, Dermatological Diseases and Conditions, Organismal Injury and Abnormalities]
8	20	14	[Amino Acid Metabolism, Cancer, Small Molecule Biochemistry]
9	18	13	[Cancer, Gastrointestinal Disease, Organismal Injury and Abnormalities]
10	18	13	[Gene Expression, RNA Damage and Repair, RNA Post-Transcriptional Modification]
11	18	13	[Cellular Compromise, Cellular Development, Cellular Growth and Proliferation]
12	18	13	[Cell Cycle, Connective Tissue Development and Function, Tissue Morphology]
13	16	12	[Cardiac Dilation, Ophthalmic Disease, Organismal Injury and Abnormalities]
14	16	12	[Hematological System Development and Function, Inflammatory Response, Tissue Morphology]
15	14	11	[Cellular Development, Cellular Growth and Proliferation, Hematological System Development and Function]
16	14	11	[Metabolic Disease, Neurological Disease, Organismal Injury and Abnormalities]
17	14	11	[Cell Cycle, Lipid Metabolism, Small Molecule Biochemistry]
18	11	9	[Hereditary Disorder, Neurological Disease, Organismal Injury and Abnormalities]
19	2	1	[Cell Cycle, Cell Morphology, Cellular Movement]
20	2	1	[Cellular Compromise, Inflammatory Response, Protein Degradation]
21	2	1	[Connective Tissue Development and Function, Organismal Injury and Abnormalities, Reproductive System Disease]

Supplementary Table S9. Top most represented diseases and biofunctions in *Mgat4d*[-/-] vs C57BL/6J *Mgat4d*[+/+] germ cells treated at 43°C. Sorted by activation Z-score.

Categories	Diseases or Functions	*p*-value	Predicted State	Activation z-score
Organismal Survival	Morbidity or mortality	1.62E-12	Increased	6.137
Organismal Survival	Organismal death	2.04E-12	Increased	6.01
Nutritional Disease,Organismal Injury and Abnormalities	Cachexia	6.88E-6	Increased	2.773
Inflammatory Response	Inflammation of body cavity	2.10E-15	Increased	2.679
Inflammatory Disease,Inflammatory Response,Organismal Injury and Abnormalities,Respiratory Disease	Inflammation of lung	1.14E-08	Increased	2.633
Infectious Diseases	Infection of mammalia	4.61E-7	Increased	2.576
Inflammatory Response,Respiratory Disease	Inflammation of respiratory system component	1.97E-12	Increased	2.549
Cardiovascular Disease,Organismal Injury and Abnormalities	Cardiac lesion	4.24E-6	Increased	2.547
Inflammatory Response,Organismal Injury and Abnormalities	Inflammation of organ	1.17E-16	Increased	2.529
Infectious Diseases	Systemic inflammatory response syndrome and/or sepsis	7.11E-08	Increased	2.414
Endocrine System Development and Function,Lipid Metabolism,Small Molecule Biochemistry,Vitamin and Mineral Metabolism	Steroidogenesis of hormone	9.86E-09	Decreased	-3.477
Lipid Metabolism,Small Molecule Biochemistry,Vitamin and Mineral Metabolism	Metabolism of terpenoid	1.37E-10	Decreased	-3.543
Cellular Movement	Invasion of cells	7.74E-08	Decreased	-3.546
Endocrine System Development and Function,Small Molecule Biochemistry	Synthesis of hormone	3.99E-08	Decreased	-3.633
Endocrine System Development and Function,Small Molecule Biochemistry	Metabolism of hormone	1.51E-7	Decreased	-3.633
Cellular Movement	Cell movement of tumor cell lines	4.27E-7	Decreased	-3.721
Organismal Development	Size of body	5.18E-09	Decreased	-3.937
Lipid Metabolism,Small Molecule Biochemistry,Vitamin and Mineral Metabolism	Synthesis of steroid	1.48E-09	Decreased	-4.209
Lipid Metabolism,Small Molecule Biochemistry	Synthesis of lipid	3.45E-12	Decreased	-4.255
Lipid Metabolism,Small Molecule Biochemistry,Vitamin and Mineral Metabolism	Synthesis of terpenoid	2.55E-10	Decreased	-4.426

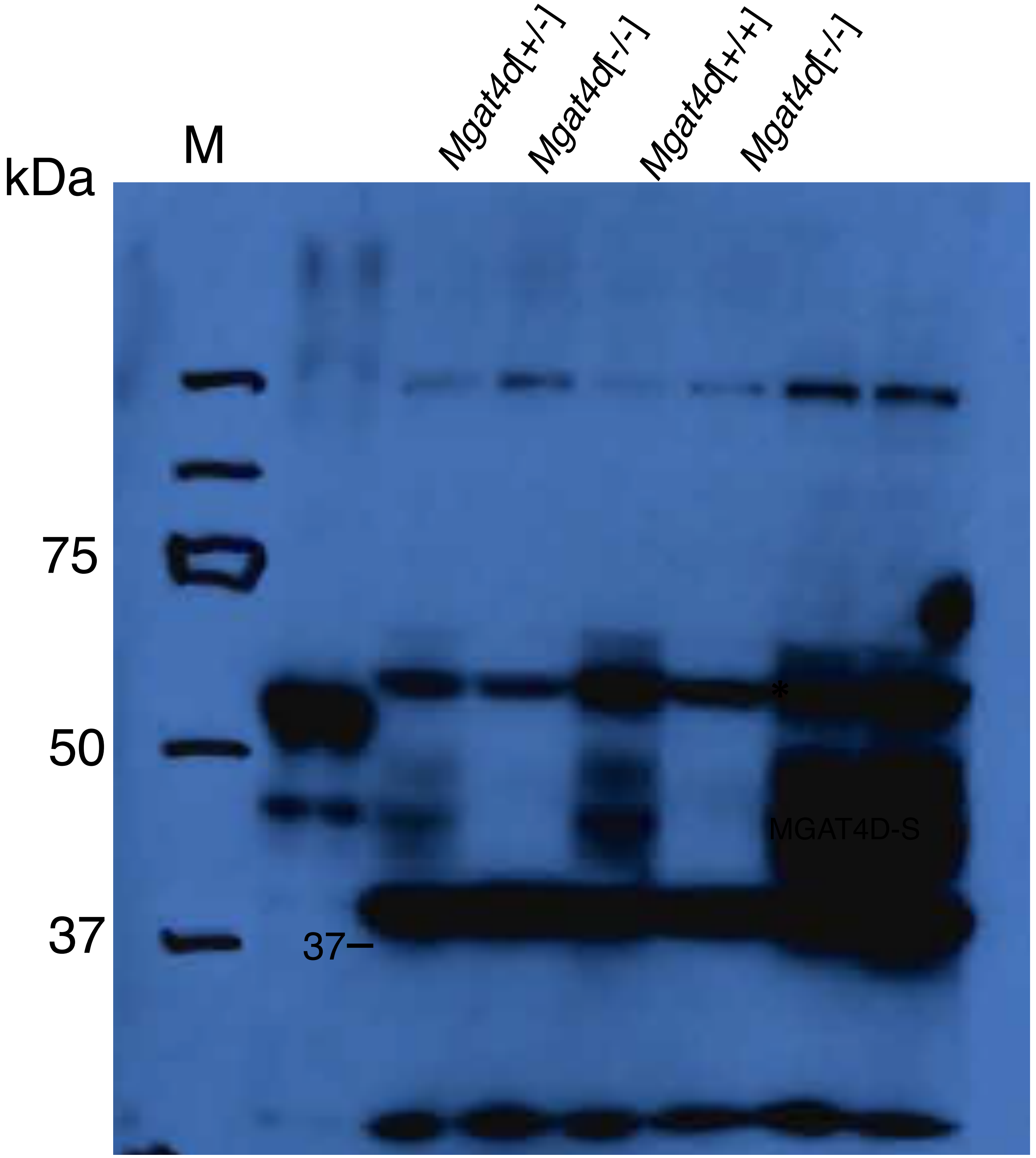

Fig. S1

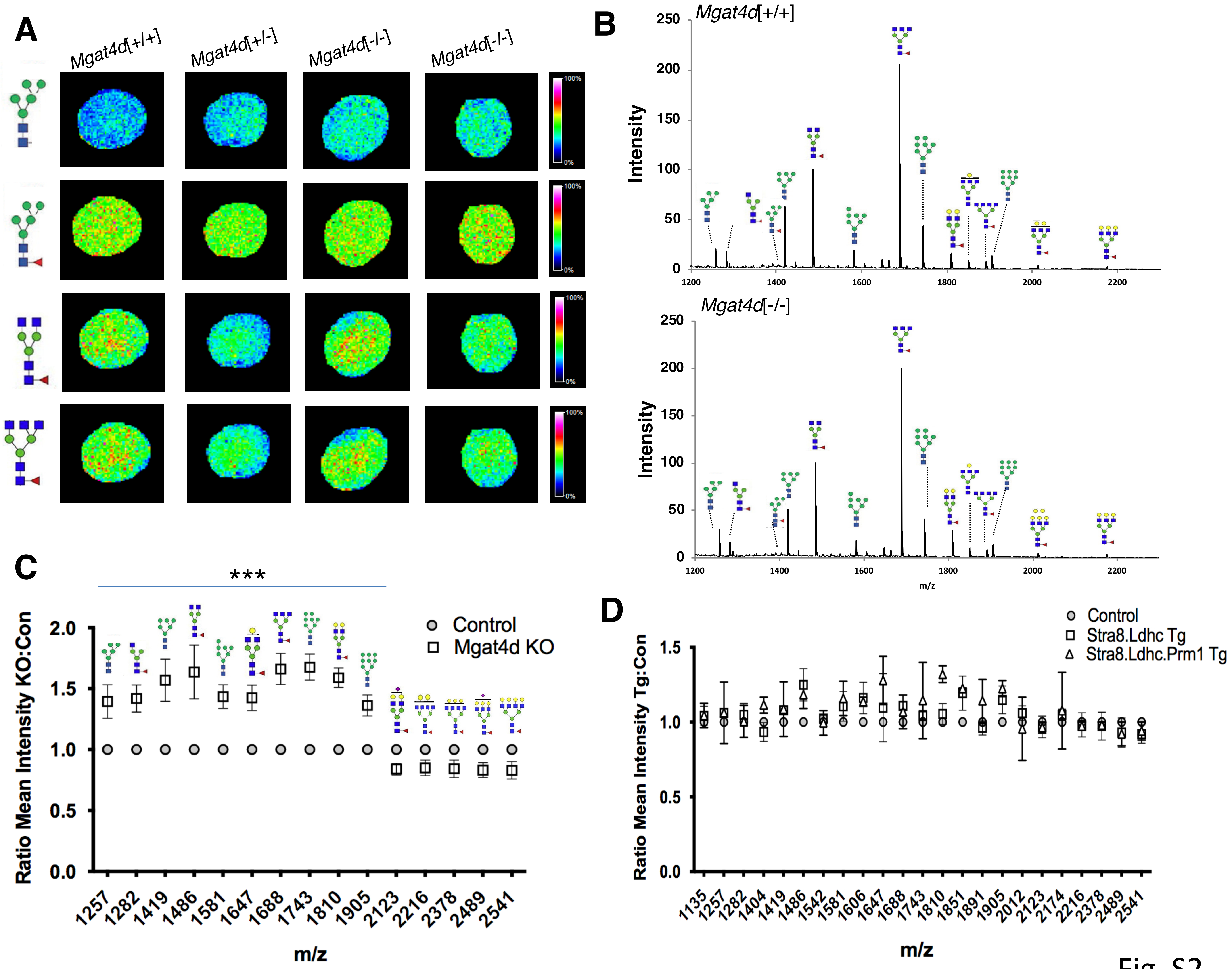
A
Mgat4d[+/+]
Mgat4d[+/-]
Mgat4d[-/-]
Mgat4d[-/-]
100%
0%
B
Mgat4d[+/+]
Mgat4d[-/-]
Intensity
m/z
C
***
Control
Mgat4d KO
Ratio Mean Intensity KO:Con
1257 1282 1419 1486 1581 1647 1688 1743 1810 1905 2123 2216 2378 2489 2541
m/z
D
Control
Stra8.Ldhc Tg
Stra8.Ldhc.Prm1 Tg
Ratio Mean Intensity Tg:Con
1135 1257 1282 1404 1419 1486 1542 1581 1606 1647 1688 1743 1810 1851 1891 1905 2012 2123 2174 2216 2378 2489 2541
m/z

Fig. S2

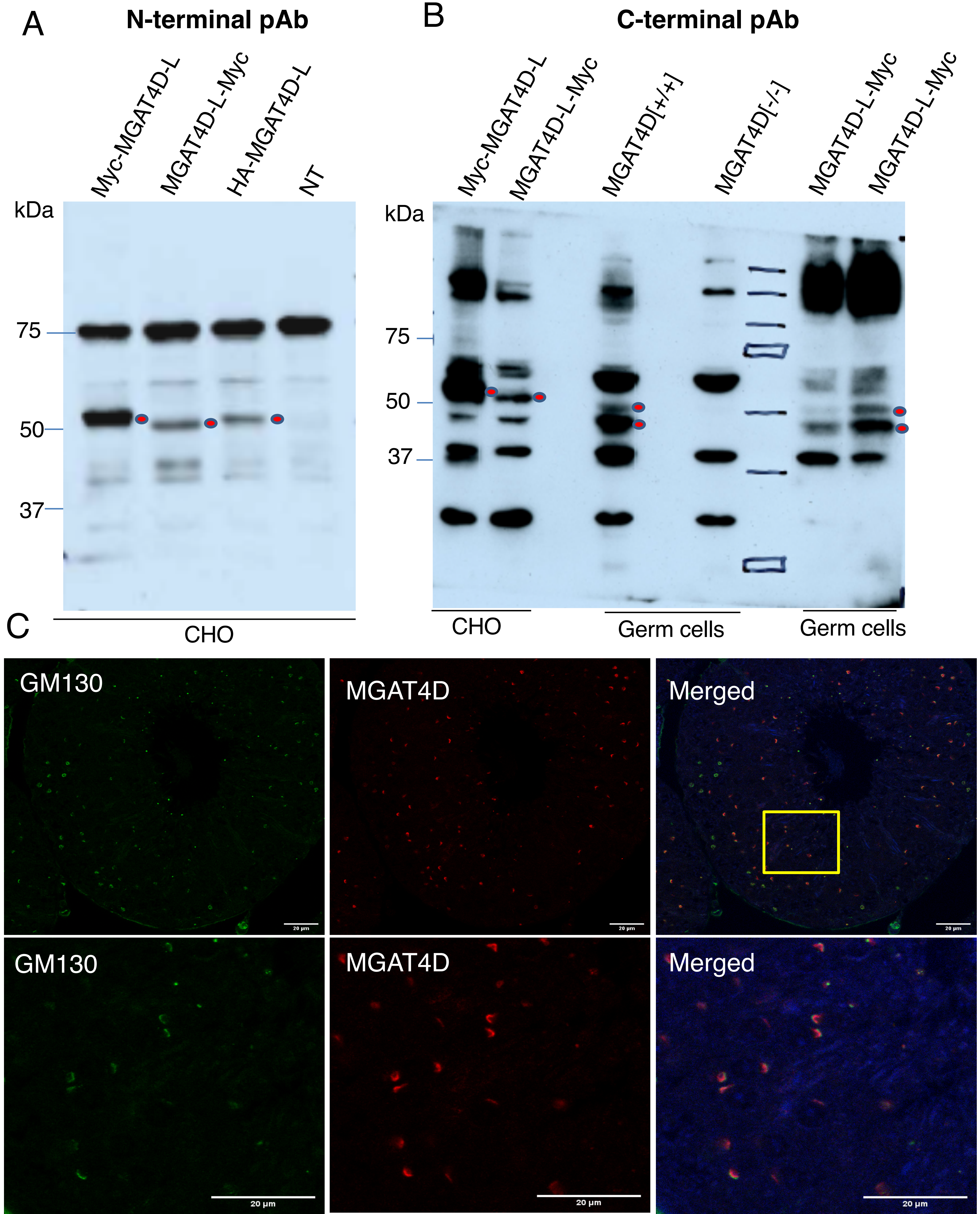
A
N-terminal pAb
Myc-MGAT4D-L
MGAT4D-L-Myc
HA-MGAT4D-L
NT
kDa
75
50
37
CHO
B
C-terminal pAb
Myc-MGAT4D-L
MGAT4D-L-Myc
MGAT4D[+/+]
MGAT4D[-/-]
MGAT4D-L-Myc
MGAT4D-L-Myc
kDa
75
50
37
CHO
Germ cells
Germ cells
C
GM130
MGAT4D
Merged
GM130
MGAT4D
Merged
20 μm
20 μm
20 μm

Fig. S3

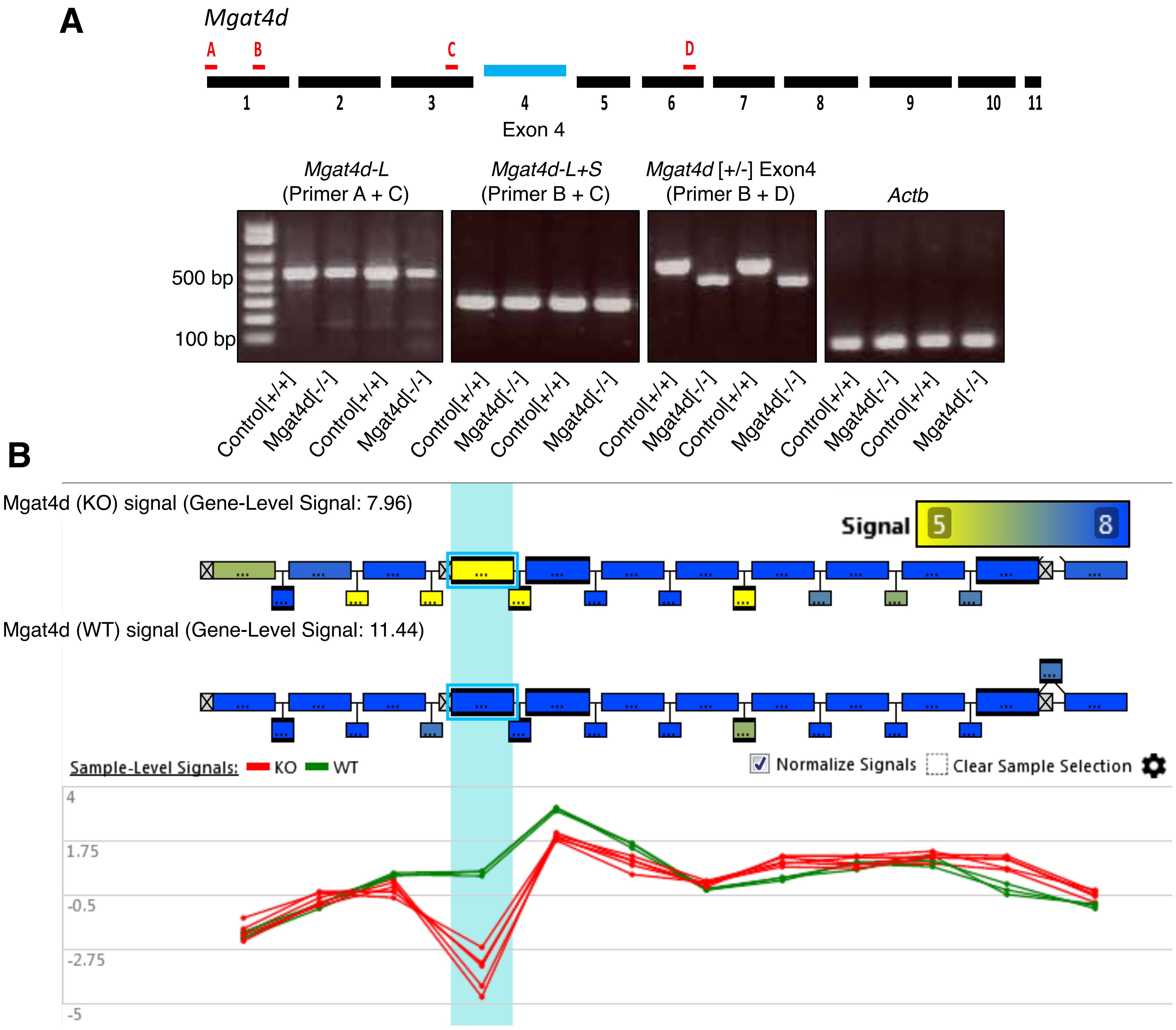
A
Mgat4d
A
B
C
D
1
2
3
4
5
6
7
8
9
10
11
Exon 4
Mgat4d-L
(Primer A + C)
Mgat4d-L+S
(Primer B + C)
Mgat4d [+/-] Exon4
(Primer B + D)
Actb
500 bp
100 bp
Control[+/+]
Mgat4d[-/-]
B
Mgat4d (KO) signal (Gene-Level Signal: 7.96)
Signal
5
8
Mgat4d (WT) signal (Gene-Level Signal: 11.44)
Sample-Level Signals:
KO
WT
Normalize Signals
Clear Sample Selection
4
1.75
-0.5
-2.75
-5

Fig. S4

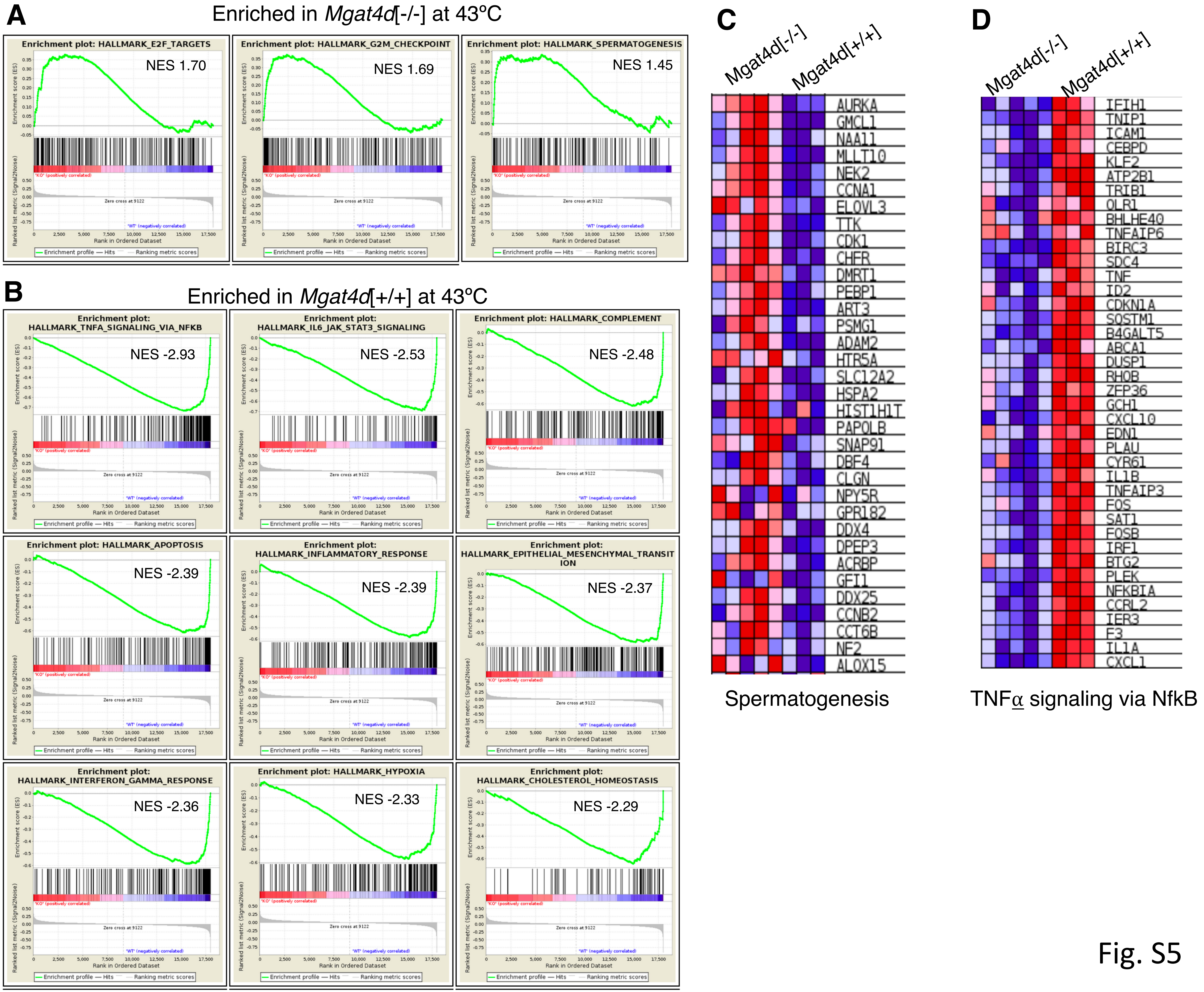
A
Enriched in Mgat4d[-/-] at 43°C
Enrichment plot: HALLMARK_E2F_TARGETS
NES 1.70
Enrichment plot: HALLMARK_G2M_CHECKPOINT
NES 1.69
Enrichment plot: HALLMARK_SPERMATOGENESIS
NES 1.45
B
Enriched in Mgat4d[+/+] at 43°C
Enrichment plot: HALLMARK_TNFA_SIGNALING_VIA_NFKB
NES -2.93
Enrichment plot: HALLMARK_IL6_JAK_STAT3_SIGNALING
NES -2.53
Enrichment plot: HALLMARK_COMPLEMENT
NES -2.48
Enrichment plot: HALLMARK_APOPTOSIS
NES -2.39
Enrichment plot: HALLMARK_INFLAMMATORY_RESPONSE
NES -2.39
Enrichment plot: HALLMARK_EPITHELIAL_MESENCHYMAL_TRANSITION
NES -2.37
Enrichment plot: HALLMARK_INTERFERON_GAMMA_RESPONSE
NES -2.36
Enrichment plot: HALLMARK_HYPOXIA
NES -2.33
Enrichment plot: HALLMARK_CHOLESTEROL_HOMEOSTASIS
NES -2.29
Enrichment score (ES)
Ranked list metric (Signal2Noise)
'KO' (positively correlated)
Zero cross at 9122
'WT' (negatively correlated)
Rank in Ordered Dataset
Enrichment profile — Hits — Ranking metric scores
C
Mgat4d[-/-]
Mgat4d[+/+]
AURKA
GMCL1
NAA11
MLLT10
NEK2
CCNA1
ELOVL3
TTK
CDK1
CHFR
DMRT1
PEBP1
ART3
PSMG1
ADAM2
HTR5A
SLC12A2
HSPA2
HIST1H1T
PAPOLB
SNAP91
DBF4
CLGN
NPY5R
GPR182
DDX4
DPEP3
ACRBP
GFI1
DDX25
CCNB2
CCT6B
NF2
ALOX15
Spermatogenesis
D
Mgat4d[-/-]
Mgat4d[+/+]
IFIH1
TNIP1
ICAM1
CEBPD
KLF2
ATP2B1
TRIB1
OLR1
BHLHE40
TNFAIP6
BIRC3
SDC4
TNF
ID2
CDKN1A
SQSTM1
B4GALT5
ABCA1
DUSP1
RHOB
ZFP36
GCH1
CXCL10
EDN1
PLAU
CYR61
IL1B
TNFAIP3
FOS
SAT1
FOSB
IRF1
BTG2
PLEK
NFKBIA
CCRL2
IER3
F3
IL1A
CXCL1
TNFα signaling via NfkB

Fig. S5
